# Suspected malignant cord compression – improving time to diagnosis via a ‘hotline’: a prospective audit

**DOI:** 10.1038/sj.bjc.6605079

**Published:** 2009-05-26

**Authors:** L Allan, L Baker, J Dewar, S Eljamel, R M Grant, J G Houston, T McLeay, A J Munro, P Levack

**Affiliations:** 1 Department of Palliative Medicine, Ninewells Hospital, Dundee DD1 9SY, Scotland, UK; 2 Department of Statistics, University of Dundee, Dundee DD1 4HN, Scotland, UK; 3 Department of Oncology, Ninewells Hospital, Dundee DD1 9SY, Scotland, UK; 4 Department of Neurosurgery, Ninewells Hospital, Dundee DD1 9SY, Scotland, UK; 5 St Brycedale Surgery, St Brycedale Road, Kirkcaldy, Scotland, UK; 6 Department of Radiology, Ninewells Hospital, Dundee DD1 9SY, Scotland, UK; 7 Department of Radiation Oncology, University of Dundee, Dundee DD1 9SY, Scotland, UK

**Keywords:** malignant spinal cord compression, rapid referral system, early diagnosis

## Abstract

The aim of the study was to achieve earlier diagnosis of malignant cord compression (MCC) using urgent magnetic resonance imaging (MRI) for selected patients. A comparison was carried out of the current prospective audit of 100 patients referred by a general practitioner or a consultant over 32 months with both a previous national Clinical Research and Audit Group (CRAG) prospective audit (324 cases of MCC) and an earlier retrospective audit of 104 patients referred with suspected MCC. A telephone hotline rapid-referral process for patients with known malignancy and new symptoms (severe nerve root pain ± severe back pain) was designed. Patients were considered for urgent MRI after discussion with a senior clinician responsible for the hotline. Appropriate referrals were discussed with radiology and oncology ensuring timely MRI reporting and intervention. The main outcome measures are as follows: time from referral to diagnosis; time from the onset of symptoms to diagnosis; and mobility at diagnosis. A total of 50 patients (52%) of those scanned had either MCC (44) or malignant nerve root compression (6) compared with the earlier rate of 23 out of 104 patients (22%). Ten out of 44 MCC patients (23%) were paralysed at diagnosis, compared with 149 out of 324 (46%) in the CRAG audit. Time from reporting pain to diagnosis was 32 days compared with 89 days in the CRAG audit. Median time from referral to diagnosis was 1 day, again considerably shorter than the CRAG audit time of 15 days (interquartile (IQ) range: 3–66). In patients at risk of MCC, fast-track referral with rapid access to MRI reduces time between symptom onset and diagnosis, improves mobility at diagnosis and reduces the number of negative MRI scans.

Numerous retrospective studies have reported that most patients with malignant cord compression (MCC) have experienced pain for a number of weeks and lost the ability to walk by the time a diagnosis is established (Bach *et al*, 1990; Larsen *et al*, 1990; Helweg-Larsen *et al*, 2000; Levack *et al*, 2001, 2002; Loblaw *et al*, 2003; Schiff 2003; Venkitaraman *et al*, 2007). The percentage of patients able to walk unaided may be as low as 18% (Levack *et al*, 2002). In addition, several large prospective studies have confirmed the dismal outcome for patients who have lost the ability to walk by the time a diagnosis is established (Helweg-Larsen and Sørensen, 1994; Makris and Kunkler 1995; Husband 1998; Rades *et al*, 2000; Baines 2002; Maranzano *et al*, 2005).

A prospective study of 153 patients with MCC reported a median survival of 3.6 months and a 1-year survival probability of 20% (Helweg-Larsen *et al*, 2000); a prospective study of two different radiotherapy schedules showed a median survival of 4 months (Maranzano *et al*, 2005); in the Scottish prospective audit of 320 patients with MCC, the median survival was 59 days (95% confidence interval: 43–75) (Levack *et al*, 2001); and in a population-based series from Canada, the median survival was 2.9 months and only 8.4% of patients were alive at 3 years (Loblaw *et al*, 2003). Despite urgent treatment, once the diagnosis of MCC has been established, many patients remain disabled and physically dependant (Larsen *et al*, 1990; Cowap *et al*, 2000; Baines 2002; Levack *et al*, 2002, 2004; Conway *et al*, 2007; Abrahm *et al*, 2008).

Data on delays in diagnosis are inevitably retrospective. Information is extracted either from hospital records or from structured interviews with patients after diagnosis. Husband (1998) reported a median length of time of 73.5 days (95% confidence interval: 60–95) between the onset of back pain and treatment. Levack *et al* (2002) reported a similar duration of symptoms in a study examining the clinical history from the separate perspectives of the hospital doctor, general practitioner (GP) and the patient. The time from the patient first reporting pain, which in retrospect was probably because of the development of MCC, to the confirmation of MCC was 90 days (interquartile (IQ) range: 37–205).

Magnetic resonance imaging (MRI) scanners have limited capacity, and urgent MRI scans should be reserved for those patients who will benefit the most or have the most to lose if diagnosis is delayed. Within the National Health Service (NHS) open access, MRI scanning is not available because of scarce resources. However, if it were possible to identify a group of patients with cancer and back pain whose symptoms were significantly more likely to be associated with epidural disease or early cord compression, then selective, urgent MRI scanning within 24 h would be justified. The clinical benefits that might be expected would be improved mobility at diagnosis, reduced dependency, reduced pain, reduction in the number of patients who require diagnosis and treatment as an in-patient and a reduced proportion of negative (for MCC) MRI studies.

If diagnosis is to be confirmed before weakness becomes permanent, then selection for scanning needs to be based on clinical symptoms or signs that occur before weakness has the opportunity to develop. Patients usually have a history of severe and worsening pain for weeks or months before the diagnosis is established (Levack *et al*, 2001; Conway *et al*, 2007). Pain reported by patients with MCC is both local and neuropathic. The former corresponds to vertebral involvement and the latter to nerve root, cord or cauda equina pressure. Untreated, continuing pressure eventually causes neurological damage and paraplegia. Most patients’ symptoms begin when they are in the community and, whether or not they are known to have cancer or not, usually present to their GP. The traditional sequence of investigation has been plain X-ray, followed by bone scan and finally by MRI, frequently not performed until the patient is unable to walk (Figure 1). Selection for MRI scanning is, however, generally poor. A retrospective review of the spinal MRI examination results of patients referred to MRI departments in Tayside for suspected cord compression, before the introduction of the hotline, reported that only 22% of referrals were confirmed on MRI imaging as MCC (Houston *et al*, 2008).

The development of nerve root pain usually prompts the patient to seek medical advice. Root pain is particularly unpleasant and is generally unresponsive to conventional analgesics. In general practice, the most likely cause of L5/S1 root pain is degenerative disc disease; hence, at the time of the study, plain X-ray of the lumbar spine was a common initial investigation. However, in patients with cancer, the development of new pain, and in particular nerve root pain, is likely to be related to tumour progression (Twycross *et al*, 1996). Furthermore, patients with epidural disease and cord compressive lesions frequently have nerve root pain that appears anatomically unrelated to the site of compression, for example, patients with thoracic epidural disease often report root pain in the anterior aspect of the leg(s). Initial assessment may be focused on the lumbar spine, leading to lumbar spine imaging in patients who ultimately prove to have thoracic MCC.

The Clinical Resource and Audit Group (CRAG) study from several Scottish centres (Levack *et al*, 2001, 2002) reported a high (84%) incidence of new, severe root pain in cancer patients who were subsequently diagnosed with MCC. It seemed reasonable, therefore, to use new, severe root pain as a trigger for further investigation in patients with cancer who, on clinical grounds, might be considered to be at high risk of MCC. Hence, using new, severe nerve root pain as a trigger in patients already known to have cancer, a rapid referral system was designed to provide urgent access to MRI scanning.

A MCC group, with representation from oncology, radiography, radiology, neurology, neurosurgery, palliative medicine, general practice and physiotherapy, was established in 2003 to re-design the service for patients with suspected MCC. The three main aims included one to improve the effectiveness of the referral system (A) and two to improve the clinical outcome (B and C) and are as follows:

- reduce time interval between a GP or a hospital doctor referral and radiological diagnosis;
- reduce the number of patients unable to walk at diagnosis; and
- reduce overall time interval between the onset of symptoms and the diagnosis of malignant cord or root compression.

We based our re-design on the lessons learnt from an earlier audit (Levack *et al*, 2001, 2002), our pooled clinical experience and on the constraints imposed by geography and available resources.

## Materials and methods

Ethics approval was obtained from the NHS Tayside Ethics Committee. A rapid referral system was designed for GPs, community nursing staff and hospital clinicians to refer patients with suspicious symptoms directly to a senior hotline clinician.

### Clinical: ‘hotline’ system

Referral criteria are as follows:

1. patient known to have, or strongly suspected to have, cancer;
2. new severe nerve root pain (unilateral or bilateral) *and/or* new severe localised vertebral pain, especially thoracic; and
3. *any* new difficulty in walking.

### Hotline process

The referring GP or a hospital doctor speaks directly with a senior clinician, currently a specialist in palliative medicine, by a dedicated phone number. After further discussion, usually between the hotline clinician and the patient's oncologist, the hotline clinician decides whether an MRI is required within 24 h. Alternatively, the hotline clinician or the patient's oncologist may arrange to examine the patient before determining whether an urgent scan is required.

An MRI slot is reserved at the end of each day's list for hotline referrals. If, by midday, the slot has not been allocated to a patient with suspected MCC, it is re-appointed to an urgent in-patient or an outpatient. Patients with probable MCC presenting after this time are scanned first thing the next morning. An *ad hoc* on-call service is available at weekends and public holidays.

**Figure 2:**
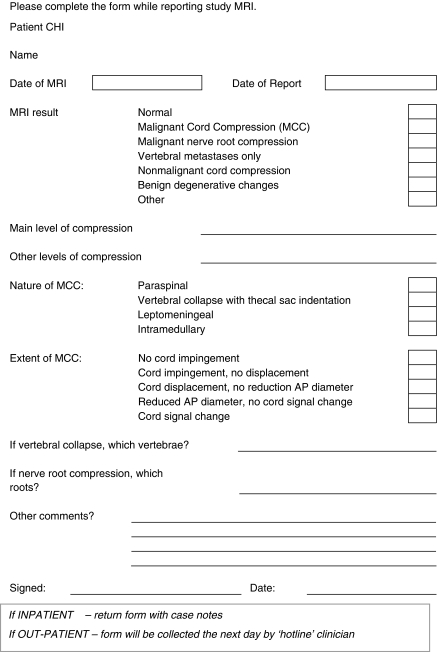
Reporting proforma for Tayside cord compression referrals.

Scans are immediately reported on a dedicated proforma (Figure 2) by radiologists with a particular interest in MRI. MRI evidence of MCC is considered to be present if there is any extension into the epidural space with impingement, displacement or compression of the cord with or without cord signal change.

The results are immediately communicated to the clinical team caring for the patient and the appropriate management is instituted.

### Data management

#### Data collection

Data were prospectively collected using a Microsoft Access database. All analyses were carried out with Minitab (Minitab Inc., State College, PA, USA, Release 14.1).

#### Data comparison

Data were compared with (a) pre-hotline analysis of referrals in Tayside for MRI for possible cord compression (unpublished audit data) and (b) CRAG data from several Scottish centres, which included information on mobility at presentation, the presence of back and neuropathic pain, duration of symptoms, underlying diagnosis and MRI findings (Levack *et al*, 2001).

## Results

Over a 32-month period, 100 patients were referred to the hotline.

A total of 16 patients were referred from their GP (either directly or by a community nurse), 16 were referred from the hospice and 68 from the hospital. Of the hospital referrals, 51 were referred by a hospital doctor or a nurse and 17 by the hospital palliative care team. In one case referred from the community, the diagnosis was suggested by an oncologist's secretary on receiving a request from a GP for an early outpatient appointment.

Underlying malignancies included lung (31), breast (13), prostate, (18), colorectal (7), upper GI (6), myeloma (6), kidney (3), lymphoma (2), other (10) and unknown (4). There were proportionally more patients with lung cancer in the hotline group (31%; 31 out of 100) than in the CRAG reference group (21%; 67 out of 319), a statistically significant difference (*P*=0.041, two-sided Fisher's exact test (2FET)). Analyses were therefore carried out for the population as a whole and for subgroups (patients with lung cancer *vs* the rest). A total of 94 patients referred to the hotline had localised back pain, 89 had nerve root pain and 84 had both.

A total of 95 patients had an urgent MRI. The results are shown in Table 1 and compared with MRI results of patients referred for imaging of possible cord compression before the hotline was introduced. Of the five patients who were referred but did not have an urgent MRI scan, one was frail and had already been treated to radiotherapeutic tolerance, the other four did not subsequently develop clinical evidence of MCC during their lifetime.

A total of 44 out of 95 patients (46.3%) referred via the hotline had MCC compared with 18 out of 104 patients (17.3%) referred before the hotline system was introduced. This is a statistically significant difference (*P*<0.0001, 2FET). If we include those with MCC or malignant nerve root compression (MNRC) in the comparison, then the rates are 23 out of 104 patients (22.1%) before the introduction of the hotline and 50 out of 95 patients (52.6%) afterwards (*P*<0.0001, 2FET).

The frequency of patients having normal or benign degenerative changes on MRI results decreased markedly on the introduction of the MCC hotline from 42.3% (pre-hotline) to 14.7% (hotline), and this change was statistically significant (*P*<0.001, 2FET). The frequency of major pathology, unrelated to cancer, seen on MRI reduced dramatically with the introduction of the hotline process from 15.4 to 0% (*P*<0.001, 2FET).

### Hotline patients with MRI diagnosis of MCC (*n*=44) or MNRC (*n*=6)

#### Outcome 1. Reduce time from a GP or a hospital doctor referral to diagnosis

The time from the GP or a hospital doctor referring the patient to the hotline, to diagnosis, was a median of 1 day (range: 0–21). One patient waited 9 days – this was a clinical decision by the palliative medicine/hotline consultant. One patient with claustrophobia needed three scans to establish a diagnosis 21 days later. Two patients waited 7 days (one had claustrophobia and the other patient's scan was delayed). This was considerably shorter than the median time of 15 days from referral to diagnosis in the CRAG audit (IQ range: 3–66 days); and significant at *P*<0.002 (Mann–Whitney *U*-test, MW) for each subgroup: overall (15 days *vs* 1 day), lung cancer (20 days *vs* 1 day) and non-lung cancer (14.5 days *vs* 1 day).

#### Outcome 2. Reduce the number of patients unable to walk at diagnosis

Of the 44 patients with proven MCC, all of whom had mobility recorded at diagnosis, 10 patients (22.7%) were unable to walk at the time of MRI diagnosis compared with 46.0% in the CRAG audit (Table 2). The overall mobility rate in the group diagnosed via the hotline is significantly better than that in the CRAG audit group (*P*=0.003, 2FET). Thirty-four patients had some mobility and 15 (34.1% of all patients with MCC) were able to walk unaided compared with only 18.8% in the CRAG audit, and once again this difference is statistically significant (*P*=0.028, 2FET). In the subgroup of patients with lung cancer, there was no statistically significant difference in the rate of ability to walk unaided (*P*=0.91, 2FET) between patients referred via the hotline and those analysed as part of the CRAG audit; this lack of statistical significance is probably because of the small numbers involved in the comparison. In the subgroup of patients who did not have lung cancer, the difference in rates was statistically significant (*P*=0.002, 2FET).

#### Outcome 3. Reduce time interval between the onset of symptoms and diagnosis of MCC or MNRC

Back pain: All 44 patients with diagnosed MCC reported back pain. For 41 of the patients, we had information on the date of onset of the back pain. The median length of time patients reported back pain before a diagnosis was established was 32 days (Table 3). This was significantly shorter than the 89 days observed in the CRAG audit (*P*=0.002, MW).

Patients with lung cancer diagnosed with MCC via the hotline had back pain for a median of 18 days, and this was significantly shorter than the length of time (113 days) for which the corresponding patients in the CRAG study experienced back pain (*P*=0.001, MW). Patients with cancers other than lung cancer had back pain for a median of 41.5 days, and although this was shorter than in the CRAG study (72 days), it did not reach statistical significance (*P*=0.255, MW).

Root pain: Forty-one out of 44 (84%) patients who had an MRI diagnosis of MCC via the Tayside hotline reported nerve root pain. For one patient no data regarding root pain were recorded, for another a report of root pain was given but no date to calculate duration of pain. The median duration of nerve root pain in the 39 patients for whom information was available was 28 days, and this compares favourably (and significantly) with a median of 89 days in the CRAG audit (*P*<0.001, MW).

In the lung cancer subgroup, the mean time from reporting root pain to diagnosis was 17.2 days in the hotline and 181.2 days in the CRAG audit, and this difference was statistically significant (*P*=0.012, two-sample *t*-test). For patients with cancers other than lung cancer, the reported median time of root pain before diagnosis was 30 days via the hotline and 72 days in CRAG, and was, again, statistically significant (*P*<0.001, MW).

### Illustrative report

An 83-year-old man with a history of prostate cancer presented to his GP with severe pain in the right renal area. A renal ultrasound showed multiple cysts in the right kidney. An intravenous urogram was subsequently performed, during which the patient experienced transient loss of sensation in his legs. On further enquiry, he had experienced a previous reaction to intravenous contrast. In view of the patient's continuing severe pain, the GP queried unilateral root pain and, bearing in mind the patient's history of cancer, telephoned the Tayside hotline. An MRI scan was carried out the same day and confirmed complete obliteration of the cord at T9. The cord compression team arranged the MRI, collected the result, discussed management with the patient's oncologist and notified the GP. The patient was treated with five fractions of radiotherapy, remained fully mobile and was discharged home after completion of the treatment.

## Discussion

### Re-designing the system

Our system shows that it is possible to identify patients who will benefit from scanning within 24 h of referral. The percentage of patients whose scans were arranged via the hotline and who turned out to have MCC or MNRC was 52%. Before the re-design, all patients in whom a diagnosis of cord compression was being queried, whether cancer-related or not, were referred for an urgent MRI. ‘Urgent’ in this context did not imply ‘immediate’, and no special measures were taken to identify or prioritise patients who might be considered, on clinical grounds, to be at particularly high risk of MCC.

With the new system, the time from the GP or a hospital doctor contacting the hotline to diagnosis of MCC or MNRC was significantly reduced, as also were the number of patients who were unable to walk at diagnosis and the duration of pain experienced before diagnosis. Knowing that carefully screened patients can be urgently scanned during the daytime reduces the need for an ‘out-of-hours’ scan.

Thus, it was possible to develop a rapid referral system for a condition, which, although uncommon in primary care, has major clinical consequences if not diagnosed. This is not the type of change that is amenable to testing in a randomised controlled trial. Nevertheless, the comparison with two different retrospective data sets provides robust evidence that the hotline system can significantly improve outcomes for patients. We had identified that rarity in clinical practice – a Pareto-type improvement. (A Pareto-type improvement is one which benefits one group of people while causing no disadvantage to any other group of people). The improved clinical results were achieved without applying any additional pressure to the MRI service.

We would suggest that a key component of this was the educational process that preceded the introduction of the hotline. By assembling a multi-professional group, we were able to contact, both directly and indirectly, those health-care professionals who would be involved in implementing the new procedures. Incorporating their views and listening to their opinions throughout the whole of the re-design process provided a two-way channel of communication: consultation and education became simply two aspects of the same enterprise. This meant that when it came to implementing the intervention, the staff involved (palliative medicine physicians, radiographers, radiologists, oncologists, clinical nurse specialists) were motivated, aware and well disposed to ensuring the success of the enterprise. This intervention was seen as an initiative developed by front-line clinicians with immediate and visible benefits for both patients and staff.

We were fortunate with our first patient referred via the hotline. This patient was walking without any help and the MRI confirmed extensive compression at T7. This helped modulate the perceptions of staff in the MRI unit. Their previous experience had led them to believe that MRI findings of MCC were invariably associated with paralysis. As a consequence, staff believed that they could make a major contribution in getting patients treated sooner. The cycle of despondency was broken. As the majority of patients accrued via the hotline were mobile, the process of scanning was physically easier, and less moving and handling were required. This contrasted with the previous experience in the scanning unit where the majority of patients with MCC had established paralysis, and all that this implies in terms of handling and dignity by the time they were scanned.

Symptoms in patients known to have active cancer have a different diagnostic interpretation than similar symptoms in patients not known to have cancer. In patients with cancer, new or escalating pain, in particular new nerve root pain, is associated with disease progression and strongly suggestive of the involvement of the spinal cord or cauda equina. One feature of the hotline system was that it afforded GPs, who may have been concerned about how to interpret worrying symptoms, the opportunity to have immediate discussions with a senior clinician and to make a plan.

Our initial efforts were concentrated on ensuring that the referral system worked smoothly within the hospital before advertising it more widely. Once the hotline was embedded within the hospital, we set out actively to increase the involvement of the wider community. A review of 42 patients subsequent to those included in this paper already shows an increase in community referrals (16 out of 42; 38%) (unpublished audit 2008).

## Conclusion

Early MRI examination and reporting is feasible for carefully selected patients. There has been minimal impact on other imaging services and no noticeable effect on overall waiting times for investigation.

More patients were referred from within hospital than from the community. However, this paper reports the first results from the hotline, and during that period, we concentrated on establishing an effective process of selection and scanning. With time, the number of referrals from the community has increased, as GPs and community-based nurses are becoming more aware of the service.

A telephone hotline offers a simple, affordable and practical method whereby MCC can be diagnosed earlier and, in this study, we have clearly shown that earlier diagnosis improves outcome, in particular patients’ mobility.

## Figures and Tables

**Figure 1:**
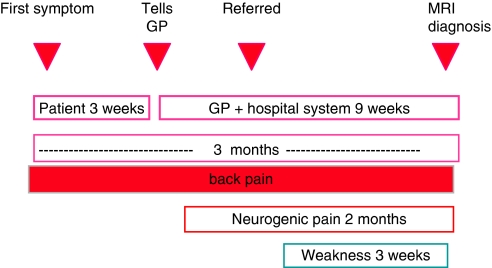
Time line of events leading to malignant cord compression from the CRAG study of several major Scottish centres 2001 (*n*=319 patients; 324 MCC episodes). Source: www.crag.scot.nhs.uk/committees/CEPS/reports/F%20Report%20copy%206-2-02.PDF.

**Table 1:** A comparison of the first 100 patients referred to the Tayside hotline and those referred over the preceding 2 years for ‘query cord compression’ (*n*=104)

	**Pre-MCC hotline^a^**			**MCC hotline**		
	***N***	**%**	***χ* ^2^**	***N***	**%**	***χ* ^2^**
Number of patients referred to MCC	104			100		
Total number of patients accessing MRI	104			95		
						
*Total number of patients accessing MRI detailed results*						
Malignant cord compression (MCC)	18	17.3	−6.4	44	46.3	+7.01
Malignant nerve root compression (MNRC)	5	4.8	−0.1	6	6.3	+0.11
Benign cord compression (BCC)	4	3.9	−0.01	4	4.2	+0.01
Vertebral metastases	17	16.4	−1.56	27	28.4	+1.71
Benign degenerative change/normal	44	42.3	+6.18	14	14.7	−6.77
Other^b^	16	15.4	+6.98	0	0.0	−7.64
						
*Total number of patients accessing MRI summary results*						
MCC or MNRC	23	22.1	−6.02	50	52.6	+6.59

MRI=magnetic resonance imaging.

a A total of 104 MRI reports of all patients with suspected cord compression between 1 January 2002 to 31 December 2003 were retrieved from the computerised Radiology Information System of Ninewells Hospital (Dundee, UK). Magnetic resonance imaging reports were re-classified according to the definitions agreed by the cord compression group to use when reporting scans from the hotline.

b Often identifying major pathology. The overall *χ*^2^ statistic is statistically significant at *χ*^2^=44.47, d.f.=5, *P*<0.001.

**Table 2:** Mobility at the time of diagnosis of MCC

	**Tayside hotline**			**CRAG audit**		
	***N***	**%**	***χ* ^2^**	***N***	**%**	***χ* ^2^**
*Overall*						
Unable to walk	10	22.7	−4.27	149	46.0	+0.58
Walking with assistance	19	43.2	+0.60	114	35.2	−0.08
Walking unaided	15	34.1	+3.85	61	18.8	−0.52
Total	44			324^a^		
						
*Lung*						
Unable to walk	4	44.4	−0.08	35	52.2	+0.01
Walking with assistance	2	22.2	−0.45	25	37.3	+0.06
Walking unaided	3	33.3	+2.78	7	10.5	−0.37
Total	9			67		
						
*Non-lung*						
Unable to walk	6	17.1	−4.89	114	44.4	+0.67
Walking with assistance	17	48.6	+1.45	89	34.6	−0.20
Walking unaided	12	34.3	+2.11	54	21.0	−0.29
Total	35			257		

CRAG=Clinical Research and Audit Group; MCC=malignant cord compression.

a *N*=319 patients, 324 separate episodes of MCC.

A comparison between the Tayside hotline and the CRAG audit.

The overall *χ*^2^ statistic is statistically significant at *χ*^2^=9.91, d.f.=2, *P*=0.007. The *χ*^2^ statistic for lung cancer patients is not statistically significant at *χ*^2^=3.76, d.f.=2, *P*=0.153, whereas the *χ*^2^ statistic for non-lung cancer patients is statistically significant at *χ*^2^=9.60, d.f.=2, *P*=0.008.

**Table 3:** A comparison of time periods (in days) between the onset of pain (back pain and nerve root pain) to diagnosis by MRI in the Tayside hotline and the CRAG audit

	**MCC hotline**			**CRAG audit**			
	***N***	**Median**	**IQ range**	***N***	**Median**	**IQ range**	***P*-value**
*Overall*							
Back pain	41	32.0	13.0–101.5	66	89.0	44.8–142.3	0.002
Root pain	39	28.0	4.0–41.0	66	89.0	44.8–142.3	<0.001
							
*Lung cancer*							
Back pain	9	18.0	5.0–37.0	13	113.0	62.5–208.5	0.001
Root pain^a^	9	17.2	6.8–27.7	13	181.2	73.8–288.6	0.012
							
*Non-lung cancer*							
Back pain	32	41.5	15.8–123.5	53	72.0	35.5–144.0	0.255
Root pain	30	29.5	5.5–51.3	53	72.0	35.5–144.0	<0.001

CRAG=Clinical Research and Audit Group; IQ=interquartile; MCC=malignant cord compression.

a With the exception of this row, all distributions are non-normal, and the median is quoted (with IQ range) with the corresponding Mann-Whitney *U*-test *P*-value. For this row, both the MCC hotline and CRAG distributions are normal, and the mean is quoted (with 95% confidence intervals) with the corresponding two-sample *t*-test *P*-value.
